# Bone health status evaluation in men by means of REMS technology

**DOI:** 10.1007/s40520-024-02728-4

**Published:** 2024-03-18

**Authors:** Adami Giovanni, Brandi Maria Luisa, Caffarelli Carla, Casciaro Ernesto, Conversano Francesco, Di Paola Marco, Fassio Angelo, Gatti Davide, Giusti Francesca, Gonnelli Stefano, Lombardi Fiorella Anna, Muratore Maurizio, Pisani Paola, Rossini Maurizio

**Affiliations:** 1 Department of Medicine, Rheumatology Unit, University of Verona, Verona, Italy; 2 Italian Bone Disease Research Foundation (FIRMO), Florence, Italy; 3 Observatory for Fragility Fractures, Florence, Italy; 4 Department of Medicine, Surgery and Neuroscience, University of Siena, Policlinico Le Scotte, Siena, Italy; 5 Institute of Clinical Physiology, National Research Council, Lecce, Italy; 6 Donatello Bone Clinic, Villa Donatello Hospital, Sesto Fiorentino, Florence, Italy; 7 ASL-LE, Ospedale Vito Fazzi, Lecce, Italy

**Keywords:** Radiofrequency Echographic Multi-Spectrometry, REMS, Men, Bone health status, Osteoporosis, Bone fragility

## Abstract

**Background:**

Osteoporosis in males is largely under-diagnosed and under-treated, with most of the diagnosis confirmed only after an osteoporotic fracture. Therefore, there is an urgent need for highly accurate and precise technologies capable of identifying osteoporosis earlier, thereby avoiding complications from fragility fractures.

**Aims:**

This study aimed to evaluate the diagnostic accuracy and precision of the non-ionizing technology Radiofrequency Echographic Multi Spectrometry (REMS) for the diagnosis of osteoporosis in a male population in comparison with conventional Dual-energy X-ray Absorptiometry (DXA).

**Methods:**

A cohort of 603 Caucasian males aged between 30 and 90 years were involved in the study. All the enrolled patients underwent lumbar and femoral scans with both DXA and REMS. The diagnostic agreement between REMS and DXA-measured BMD was expressed by Pearson correlation coefficient and Bland-Altman method. The accuracy of the diagnostic classification was evaluated by the assessment of sensitivity and specificity considering DXA as reference.

**Results:**

A significant correlation between REMS- and DXA-measured T-score values (*r* = 0.91, *p* < 0.0001) for lumbar spine and for femoral neck *(r* = 0.90, *p* < 0.0001) documented the substantial equivalence of the two measurement techniques. Bland-Altman outcomes showed that the average difference in T-score measurement is very close to zero (−0.06 ± 0.60 g/cm^2^ for lumbar spine and − 0.07 ± 0.44 g/cm^2^ for femoral neck) confirming the agreement between the two techniques. Furthermore, REMS resulted an effective technique to discriminate osteoporotic patients from the non-osteoporotic ones on both lumbar spine (sensitivity = 90.1%, specificity = 93.6%) and femoral neck (sensitivity = 90.9%, specificity = 94.6%). Precision yielded RMS-CV = 0.40% for spine and RMS-CV = 0.34% for femur.

**Conclusion:**

REMS, is a reliable technology for the diagnosis of osteoporosis also in men. This evidence corroborates its high diagnostic performance already observed in previous studies involving female populations.

## Introduction

Osteoporosis is a systemic skeletal disease characterized by low bone mass and microarchitectural deterioration, with an increase of fracture risk [1]. Thought gender differences in osteoporosis exist, for long time this pathology has been regarded as a typical and almost exclusive female condition [2].

Globally, the prevalence of osteoporosis in women accounts for 23%, whereas in men is reported to be nearly 12% [3]. In Europe plus Switzerland and the United Kingdom, in 2019 osteoporosis and its consequences involved 25.5 million women and 6.5 million men, with 4.3 million people suffering from fragility fractures [4, 5]. In U.S. in 2014 about 10 million people aged 50 years and older suffered from osteoporosis [6]; its prevalence is higher in women than in men, and higher in individuals aged 65 years and older than in adults aged 50–64 years [7]. In Asia Pacific osteoporosis affects 10–30% for women aged 40 and older, and up to 10% for men [8].

Despite these estimates showed that osteoporosis is more prevalent in women than in men, with a female-ratio of about 4, on the other hand, in about 30–40% of new osteoporotic fractures worldwide have occurred in men, with a female-male ratio of 1.6, thereby suggesting that osteoporosis in men requires clinical attention [9, 10]. In general, men are more susceptible to traumatic fractures in the age range of 18–45 years and to osteoporotic fractures over the age of 75, where a densitometry test is recommended [10]. Since osteoporosis is generally recognized as a “silent disease”, causing no symptoms until a fracture or vertebral collapse occur, it appears clear that male osteoporosis is largely under-diagnosed and under-treated due to low screening rates, with most of the diagnosis confirmed after an osteoporotic fracture. Furthermore, in order to understand the impact of this problem, it should be taken into account that the mortality rate associated with major osteoporotic fractures is higher in male patients than females, with the former having more osteoporosis-related complications [10, 11]: in older men, the mortality rate due to osteoporotic hip fracture is twice that of women [12].

The diagnosis of osteoporosis is currently established by the measurement of bone mineral density (BMD) in correspondence of the major sites prone to fractures, such as the spine and hip, or following the occurrence of femoral or vertebral fracture in the absence of major trauma [13, 14]. Currently, Dual X-ray Absorptiometry (DXA) of proximal femur and lumbar spine is deemed as the standard technology to establish a diagnosis of osteoporosis, despite the known limitations of this technology (the use of ionizing radiations, measurement inaccuracies due to the presence of artefacts associated to bone deformities, calcifications, or fractures along with the lack of standardization across manufacturers, etc. [15, 16]. . Clinical routine DXA scans are often performed with a poor adherence to the guidelines of the International Society for Clinical Densitometry (ISCD) [13, 17, 18], with the result that more than 90% of DXA reports presented operator-dependent errors [15, 19].

A possibility to overcome DXA limitations might be represented by the Radiofrequency Echographic Multi Spectrometry (REMS) technology, a non-ionizing approach for osteoporosis diagnosis based on an ultrasound acquisition of femoral neck and/or lumbar spine. Each scan lasted 80 s and 40 s for lumbar spine and femoral neck examination, respectively, and is followed by an automatic processing time of about 1–2 min [20]. The average cost for REMS examination is about €80,00 as shown in the Health Technology Assessment (HTA) study of REMS in the diagnosis of osteoporosis [21]. A dedicated rigorous training is provided to the operator for the use of the device implementing REMS technology. REMS technology has been clinically validated through multi-center clinical studies [22–24] and presented in a consensus paper by the European Society for Clinical and Economic Aspects of Osteoporosis, Osteoarthritis and Musculoskeletal Diseases (ESCEO) as a valuable technology for osteoporosis diagnosis and fracture risk estimation [25]. The BMD assessed by REMS has shown significant correlations with the corresponding BMD values measured by DXA. A high agreement between REMS-based and DXA-based diagnoses has been demonstrated when both examinations are correctly performed according to the guidelines, with the capacity to discriminate osteoporotic subjects from non-osteoporotic ones [22, 23] and good performance in the identification of patients with or without incident fragility fractures [24]. These validation studies involved female patients, including postmenopausal women aged 51 to 70 [22] or women covering a wide age range from 30 to 90 [23, 24]. Nevertheless, no study has evaluated the diagnostic performance of REMS in the male population until now.

The aim of this multicenter cross-sectional observational study was to assess the short-term precision, inter-operator repeatability and diagnostic accuracy of lumbar and femoral REMS investigations in a population of male subjects in comparison with the outcome of the DXA, when both examinations have been conducted in accordance with the relevant user manuals or guidelines.

## Materials and methods

The clinical data were collected through a multicenter cross-sectional observational study on male patients fulfilling the following enrollment criteria: Caucasian ethnicity, age range between 30 and 90, body mass index (BMI) including normal- or under-weight, overweight and obese, absence of significant walking impairments, medical prescription for a spinal and/or femoral DXA investigation. The patients were recruited from 4 Italian centers: “Galateo” Hospital (San Cesario di Lecce, Lecce), “Le Scotte” University Hospital (Siena), “Careggi” University Hospital (Florence), “Borgo Roma Gianbattista Rossi” University Hospital (Verona). For all the participants, the BMD was measured at the lumbar spine and femoral neck by a DXA investigation (according to their medical prescription) and an echographic scan by REMS. The study protocol was approved by the Ethics Review Boards of all the participating hospitals. All the enrolled patients voluntarily entered the study after giving written informed consent.

### DXA measurements

DXA scans were performed according to the standard clinical routine procedures. When measuring a patient, spinal investigations were carried out with hip and knee both at 90° of flexion, whereas for femoral examinations the patient’s femur was straight on the table, such that the shaft was parallel to the vertical edge of the obtained image, and with 15°–25° of internal rotation achieved by using a dedicated positioning device. All the DXA medical reports were anonymized before subsequent analyses.

### REMS acquisitions

REMS scans of lumbar vertebrae and proximal femur were performed using the EchoStation device (Echolight Spa, Lecce, Italy), equipped with a convex transducer operating at the nominal frequency of 3.5 MHz. For each acquisition, the final medical report, the corresponding sequence of B-mode images and the unprocessed raw ultrasound signals were automatically stored.

For each vertebral or femoral patient acquisition, the operator set the transducer focus and scan depth in order to obtain the target bone interface (i.e., lumbar vertebra surface or femoral neck profile) in the ultrasound beam focal zone, so as to visualize it approximately in the middle of the B-mode image and at a distance of at least 3 cm from the bottom image. In particular, for a lumbar spine scan, the operator placed the echographic transducer trans-abdominally under the sternum, in order to center the L1 vertebra profile in the middle of the B-mode reconstructed image and then moved the transducer across L2, L3 and L4 according to the on-screen and audio guided indications provided by the device software. For a femoral neck scan, the operator held the echographic transducer parallel to head-neck axis of the femur, as to visualize the proximal femur profile, including the interfaces of femoral head, neck and trochanter in the field of view of the B-mode image.

At each clinical center participating in the study, the data collected from the first 10 enrolled patients who underwent two consecutive REMS scans on each considered anatomical site, were used to assess both the intra- and inter-operator repeatability of BMD measurements.

Intra-operator repeatability (also referred to as short-term precision) was assessed on two consecutive measurements on the first 8 patients by an experienced operator. Inter-operator repeatability was evaluated on each one of the subsequent 8 patients, for whom two consecutive measurements were performed by two different operators: an experienced one and another one who had previously received a 3-h training session only. All the remaining patients underwent a single scan on each considered anatomical site with the aim to evaluate the intrinsic diagnostic accuracy of the REMS technology. All the REMS medical reports, together with the corresponding echographic images and related raw signals, were anonymized before starting the subsequent analyses.

## Data analysis

### Short-term precision

According to the method described by Di Paola et al. [22], intra-operator repeatability was assessed in terms of “short-term precision” as defined by Engelke and Gluer [26], using the data acquired on the first 8 male patients enrolled in each of the 4 clinical centers involved in the study and a total of 32 cases was included in the analysis. As recommended by the International Society of Clinical Densitometry (ISCD) guidelines, the standard deviation (SD) of repeated BMD_US_ measurements was calculated for each patient and the precision was expressed as the root-mean-square coefficient of variation (RMS-CV); the least significant change (LSC) at 95% confidence level and smallest detectable difference (SDD) were also calculated.

### Inter-operator repeatability

As described by Di Paola et al. [22], for each considered anatomical site, inter-operator repeatability was assessed on the data acquired on the second set of 8 male patients enrolled in each of the 4 clinical centers involved in the study and a total of 32 cases was included in the analysis. Calculations were carried out similarly to those for short-term precision and the inter-operator repeatability was expressed in terms of RMS-CV and LSC at 95% confidence interval.

### Diagnostic accuracy

The analysis of the medical reports was performed separately for lumbar spine and femoral neck sites, both for DXA and REMS acquisitions. Using T-score values obtained from DXA and REMS measurements on each anatomical region, every patient was classified as “osteoporotic” with T-score ≤-2.5 and as “non-osteoporotic” with T-score ˃-2.5. Furthermore, the “non-osteoporotic” group of patients was further classified as “osteopenic” if -2.5 < T-score <-1.0 and/or “healthy” with T-score ≥-1.0. According to the rigorous approach described by Di Paola et al. [22], in order to assure the maximum reliability of the diagnostic outputs, all the collected medical reports (DXA and REMS), with the corresponding B-mode ultrasound images and raw data in the REMS case, were independently checked by two experienced operators in order to identify any possible errors that could have provided improper measurements, potentially resulting in inappropriate diagnostic classifications. DXA errors were identified according to the ISCD guidelines [13] and to the indications from recent literature [15]: they were typically associated with inaccurate patient positioning, wrong data analysis (e.g., incorrect placement of analysis boxes in the image), presence of artifacts, or mistakes in the input of demographic characteristics.

Instead, REMS errors were identified as deviations from the acquisition procedure described in the EchoStation user manual: they were typically associated with wrong or suboptimal settings of transducer focus and/or scan depth, or with incomplete adherence to the on-screen and audible indications provided by the software (e.g. missing or delayed movement from a given vertebra to the subsequent one).

Both operators, who were blind with respect to each other findings, were asked to carefully check each medical report for the possible presence of any of the previously listed error types. For instance, referring to wrong data analyses associated with DXA scans, a typical error was represented by a slight misplacement (1–2 mm) of an intervertebral line or by the inclusion in the analysis of lumbar vertebrae not belonging to L1-L4. On the other hand, referring to REMS acquisitions, considering that transducer focus could be set only at fixed values (e.g., 21 mm, 36 mm, 45 mm, 53 mm, etc.), a typical error was the selection of a transducer focus different from the “ideal” value. Once both the operators independently completed the report analysis, they discussed together all the cases that had received different classifications (i.e., presence/absence of errors and/or type of error) until a consensus was reached.

For each considered anatomical site, only DXA and REMS reports containing errors were excluded from subsequent analyses.

Diagnostic accuracy of the REMS approach was then assessed on the remaining patients by assuming DXA outputs as the gold standard reference and by determining sensitivity and specificity in the discrimination between “osteoporotic” and “non-osteoporotic” patients.

The diagnostic concordance between DXA and REMS methods was also assessed, by calculating the percentage of patients classified in the same diagnostic category (osteoporotic, osteopenic or healthy) and the corresponding Cohen’s kappa (k). The analysis in terms of diagnostic classification was performed independently for lumbar spine and femoral neck sites, both for DXA and REMS acquisitions. In order to assess the concordance in 3 diagnostic classes (from here on referred to as “diagnostic concordance”) between the two densitometric technologies, each patient was classified as osteoporotic if T-score was ≤ − 2.5, osteopenic if − 2.5 < T-score < − 1.0 or healthy if T-score ≥ − 1.0.

Moreover, in order to take the borderline cases into account, namely misclassifications deriving from T-score values that stand at the limit of the typical threshold values of -2.5 and − 1, the accuracy and diagnostic agreement parameters were recalculated accepting a 0.3 tolerance on the T-score of these borderline cases, according to an approach already adopted in previous studies [22, 24].

Furthermore, the degree of correlation between DXA and REMS T-score values was quantified through a linear regression analysis, by calculating the slope of the regression line, the Pearson’s correlation coefficient (r) and the coefficient of determination (r^2^). Finally, we directly assessed the agreement between T-score values measured by REMS and DXA by measuring the standard error of the estimate (SEE) and through the Bland-Altman method [27].

In order to compare BMD with BMD_US_ values, we had to take into account that there are systematic differences in how BMD values are measured and reported among DXA scanners from various manufacturers. Since BMD_US_ already showed a very good correlation with BMD measurements performed with Hologic densitometers [28, 29], BMD values measured by Lunar scanners were preliminarily converted in Hologic-equivalent values by applying specific conversion formulas derived from literature for both lumbar spine [30] and femoral neck [31].

## Results

### Study population and preliminary medical report analysis

According to their medical prescription, a total of 603 men aged between 30 and 90 years included in this study underwent only lumbar investigations, whereas 587 were examined only on the femoral site. Based on BMI values, 256 (42.4%) the patients in the lumbar group were normal- or under-weight, 250 (41.5% were overweight, and 97 (16.1%) were obese; in the femoral group, we had 243 (41.4%) normal- or under-weight, 261 (44.5%) overweight, and 83 (14.1%) obese patients.

Table 1 summarizes the average patient characteristics for each considered anatomical site, together with the results of the quality assessment on medical reports, which resulted in the exclusion of: (i) 53 (8.8%) patients from the lumbar group because of DXA errors and 42 (7.0%) because of REMS errors, respectively; (ii) 44 (7.5%) patients from the femoral group due to DXA errors and 31 (5.3%) to REMS errors, respectively.

As a result, the REMS diagnostic accuracy was assessed considering 508 patients for lumbar spine and 512 patients for femoral neck. The percentages of underweight, normal-weight, overweight and obese patients was substantially unchanged for both the lumbar group and the femoral group.

**Table 1:** Average characteristics of the enrolled patients for each considered anatomical site and results of the quality assessment on medical reports

	Lumbar spine	Femoral neck
Enrolled patients	603	587
Age (y)	58.3 ± 14.5	58.9 ± 14.4
Height (cm)	172.6 ± 7.8	172.2 ± 7.7
Weight (kg)	78.1 ± 13.0	77.8 ± 12.9
BMI (kg/m^2^)	26.2 ± 4.0	26.2 ± 4.1
Excluded DXA reports	53 (8.8%)	44 (7.5%)
Wrong data analysis	11 (1.8%)	19 (3.2%)
Inaccurate patient positioning	28 (4.6%)	18 (3.1%)
Presence of artifacts	9 (1.5%)	2 (0.3%)
Data input mistakes	5 (0.8%)	5 (0.9%)
Excluded REMS reports	42 (7.0%)	31 (5.3%)
Wrong focus selection	23 (3.8%)	18 (3.1%)
Wrong scan depth selection	13 (2.2%)	9 (1.5%)
No adherence to scan procedure	6 (1.0%)	4 (0.7%)

### Diagnostic accuracy of the REMS approach implemented in the EchoStation device

**Fig. 1:**
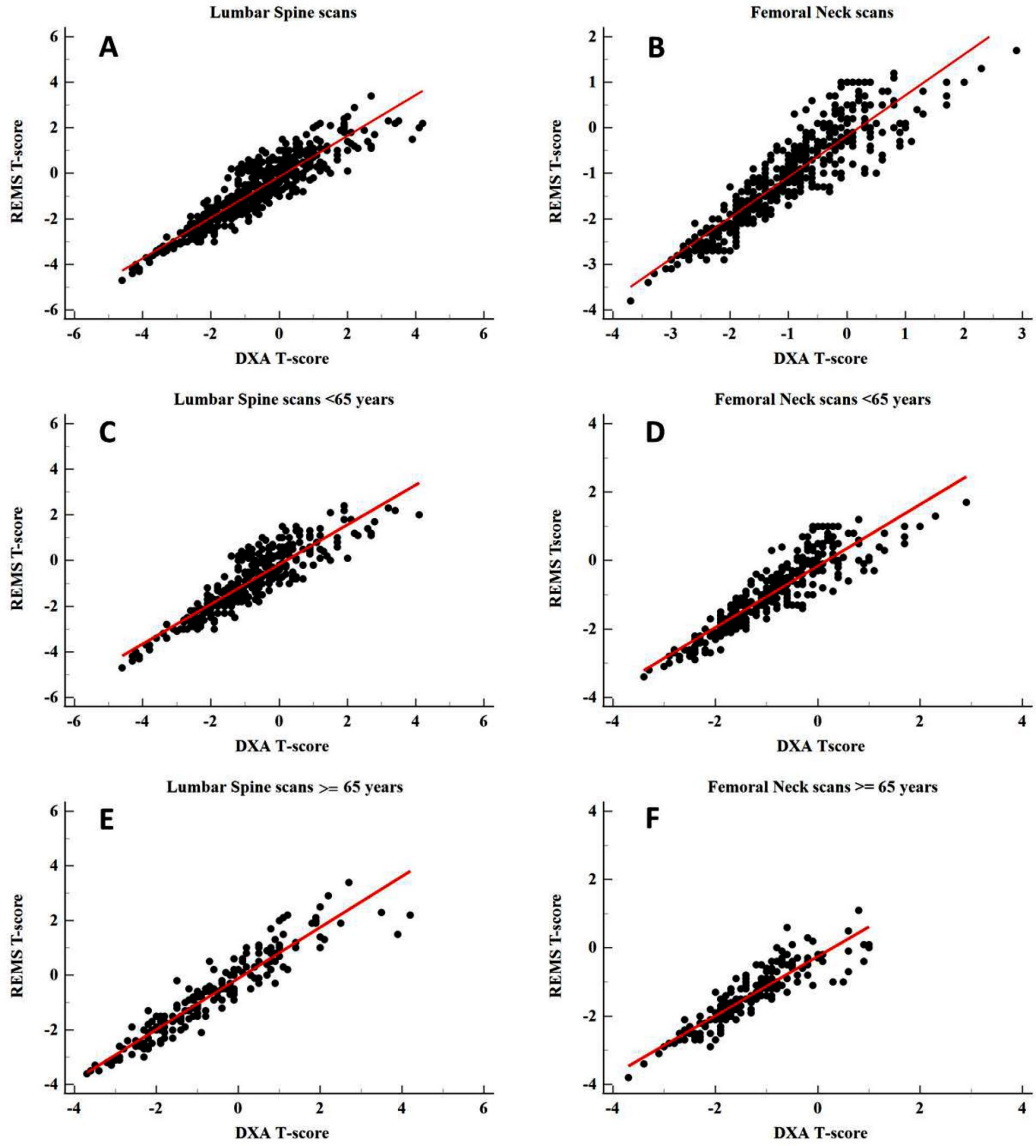
Scatterplot of DXA T-score and REMS T-score. In panel **A**, **C** and **E**, values obtained from lumbar spine scans are shown: the slope of the regression line is 0.91 for the total population, 0.89 for group A and 0.94 for group B, Pearson correlation coefficient *r* = 0.91 (*p* < 0.0001) for the total population, 0.89 (*p* < 0.0001) for group A and *r* = 0.94 (*p* < 0.0001) for group B; in panel **B**, **D** and **F**, values obtained from femoral neck scans are shown, with slope of the regression line 0.90 for the total population, 0.90 for group A and 0.90 for group B, Pearson correlation coefficient *r* = 0.90 (*p* < 0.0001) for the total population, 0.90 (*p* < 0.0001) for group A and 0.90 (*p* < 0.0001) for group B

Considering the T-score values provided by the two techniques, a high degree of correlation was found for both lumbar spine (*r* = 0.91, *p* < 0.0001) and femoral neck (*r* = 0.90, *p* < 0.0001). The slopes of the corresponding regression lines were 0.91 for spine and 0.90 for femur, as shown in Fig. 1A and B, respectively. Moreover, a high correlation was also found when patients were divided into two subgroups, < 65 years old (group A) and ≥ 65 years old (group B). For lumbar spine a correlation equal to *r* = 0.89 (*p* < 0.0001) and *r* = 0.94 (*p* < 0.0001) was obtained for group A for group B, respectively. Similar results were found for the femoral neck (*r* = 0.90, *p* < 0.0001 for group A and *r* = 0.90, *p* < 0.0001 for group B). The regression lines slopes for spine were 0.89 (group A) and 0.94 (group B), while for femoral neck were 0.90 (group A) and 0.90 (group B) as shown in Fig. 1C–F.

The REMS approach discriminated the osteoporotic patients from the non-osteoporotic ones with a sensitivity of 90.1% and a specificity of 93.6% using lumbar spine scan, among all the enrolled subjects. Correspondingly, for femoral neck scans, a sensitivity of 90.9% and specificity of 94.6% were found considering all subjects. Subsequently, the sensitivity and specificity for the group A and B both for lumbar spine and femoral neck were calculated. For the lumbar spine the sensitivity for group A and B was 91.4% and 85.0%, respectively; when the 0.3 T-score tolerance was considered the sensitivity increased to 100% and 95.0%. For the femoral neck the sensitivity in both groups was 100% (group A) and 81.3% (group B); with the 0.3 T-score tolerance the sensitivity increased to 100% and 93.8%, respectively. Moreover, the specificity for lumbar spine for group A and B was 94.5% and 92.0%, respectively, and increased to 98.6% and 99.4%, respectively, with the 0.3 T-score tolerance. For femoral neck the specificity for both groups was 95.7% (group A) and 92.5% (group B), respectively, and increased to 99.7% for group A and 99.4%, for group B when the 0.3 T-score tolerance was considered.

When all the three diagnostic classes (osteoporotic, osteopenic and healthy) were considered, classification agreement between DXA and REMS was found in 82.7% of cases (K = 0.71, *p* < 0.001) for lumbar spine and 81.8% (K = 0.71, *p* < 0.001) for femoral neck, respectively. With the 0.3 T-score tolerance, the diagnostic concordance increased to 92.3% and 95.7% for lumbar spine and for femoral neck, respectively. Similar classification agreement was found when the population was divided into group A and group B. In particular for lumbar spine was found in 82.8% (K = 0.67, *p* < 0.001) of cases for group A and 82.5% of cases (0.79, *p* < 0.001) for group B; for femoral neck was found in 84.5% (K = 0.70, *p* < 0.001) for group A and 77.4% (K = 0.70, *p* < 0.001) for group B. When the 0.3 T-score tolerance was considered, the diagnostic concordance in both groups A and B increased to 91.0% and 94.5% for lumbar spine; and to 95.7% and 95.8% for femoral neck.

**Fig. 2:**
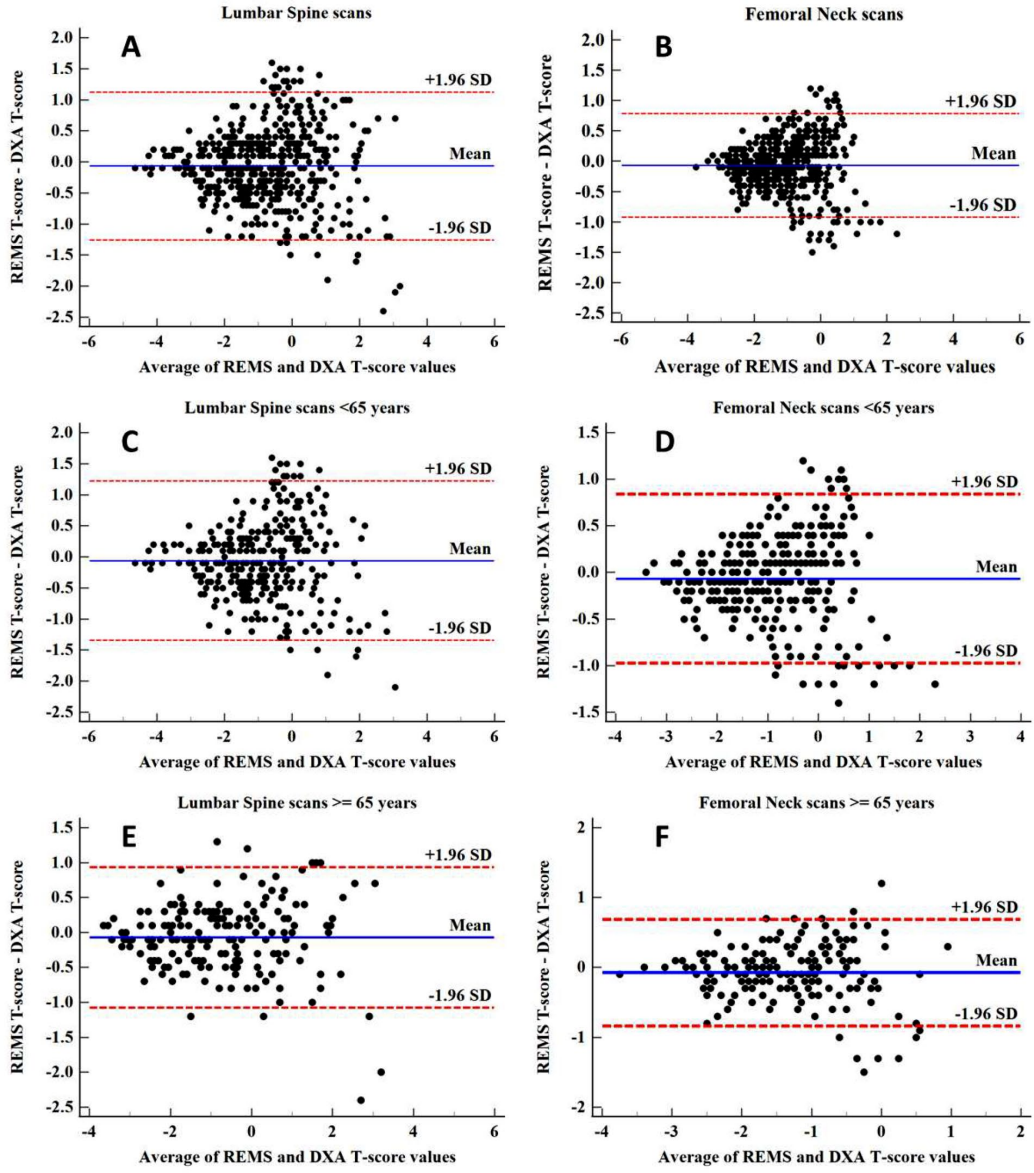
Bland-Altman plot comparing REMS and DXA T-score measurements for lumbar spine site (panel **A**, **C** and **E**) and femoral neck site (panel **B**, **D** and **F**)

The Bland-Altman plots showing the differences between DXA- and REMS-measured T-score values for each considered anatomical site are reported in Fig. 2: the average difference (expressed as bias ± 1.96 SDs) was − 0.06 ± 0.60 g/cm^2^ for lumbar spine (Fig. 2A) and − 0.07 ± 0.44 g/cm^2^ for femoral neck (Fig. 2b). Even when the population was divided into the two groups for age, A (< 65 y) and B (≥ 65 y), analogous Bland-Altman values were found for both lumbar spine (-0.06 ± 0.65 g/cm^2^ for age < 65 y, -0.07 ± 0.51 g/cm^2^ for age ≥ 65 y) and femoral neck (-0.07 ± 0.46 g/cm^2^, -0.07 ± 0.84 g/cm^2^). The Bland-Altman plots also emphasize the absence of any visible trend of the difference between REMS and DXA-measured T-score values to their average value, suggesting that REMS accuracy in the estimation of T-score does not depend on the size of the T-score value itself. These results, combined with the values of the coefficient of determination (r^2^ = 0.83 for the total population, r^2^ = 0.79 for group A, r^2^ = 0.89 for group B for lumbar spine; and r^2^ = 0.82 for the total population, r^2^ = 0.81 for group A, r^2^ = 0.81 for group B for femoral neck) and with the corresponding standard errors of the estimate (SEE = 0.045 g/cm^2^ for the total population, SEE = 0.041 g/cm^2^ for group A, SEE = 0.041 g/cm^2^ for group B for lumbar spine; and SEE = 0.031 g/cm^2^ for the total population, SEE = 0.033 g/cm^2^ for group A, SEE = 0.049 g/cm^2^ for group B for femoral neck) documented the actual strength of the relationship between BMD_US_ and BMD. Table 2 summarizes the results obtained with REMS for each of the considered anatomical sites.

**Table 2:** Results of the REMS accuracy evaluations for each considered anatomical site. In all the calculations, DXA results obtained for the retained patients after the exclusion of medical reports containing errors were assumed as the reference ground truth

Anatomical site	Lumbar spine	Femoral neck
Retained cases (n)	508	512
Sensitivity	90.1%	90.9%
Specificity	93.6%	94.6%
Diagnostic concordance	82.7%	81.8%
*K*	0.71*	0.71*
*r*	0.91*	0.90*
*r* ^*2*^	0.83*	0.82*
Regression line slope	0.90	0.90
SEE (g/cm^2^)	0.045	0.031
Average difference (bias ± 1.96 SD, g/cm^2^)	−0.06 ± 0.60	−0.07 ± 0.44

*p < 0.0001

### REMS precision and repeatability

Short-term precision, expressed as RMS-CV, was 0.40% for lumbar spine and 0.34% for femoral neck, and the corresponding LSC value at 95% confidence level was 1.11% for lumbar spine and 0.95% for femoral neck, respectively. The SDD value resulted to be 0.010 g/cm^2^ for lumbar spine and 0.008 g/cm^2^ for femoral neck.

Analogous calculations were performed to assess inter-operator variability, producing the following results: RMS-CV = 0.57% and LSC = 1.57% for lumbar spine; RMS-CV = 0.52% and LSC = 1.43% for femoral neck.

## Discussion

This multicenter clinical study assessed the diagnostic performance of REMS investigations in a male population, considering DXA as reference, and respecting all the requirements of good practice for either techniques, in order to avoid errors that could affect the final outcome. The accuracy of the REMS technology for bone health assessment has already been largely demonstrated in a female population [20]. In Pisani et al. [32], the capability of REMS technology for the prediction of incident fragility fractures at 5 years by Fragility Score (FS) indicator was evaluated in a male population where FS has been shown to effectively identified patients at risk for incident fragility fractures showing the highest prediction ability for any considered fracture type (AUC = 0.780 and AUC = 0.809 for generic osteoporotic fractures and hip fractures, respectively), resulting always significantly higher than either T-scores measured by DXA and REMS. The population investigated here, whose age ranged between 30 and 90 years reflects the opportunity for males, both young and elderly, to benefit from a safe ionizing radiation-free approach for the diagnosis of osteoporosis and bone health assessment on the axial reference anatomical sites. The two limitations of this study involve the following aspects: the population entirely composed of Caucasian ethnicity, and no patients with secondary osteoporosis were enrolled.

The results obtained from the present study demonstrated that, if the guidelines and recommendations are scrupulously followed, the actual diagnostic capabilities of the REMS approach result in both sensitivity and specificity above 90% for each considered anatomical site for the total considered population. This result is particularly relevant, since both the ISCD [9, 25] and the UK National Osteoporosis Society (NOS) [34] recommend the use of device specific upper and lower thresholds, with 90% sensitivity and 90% specificity, to identify patients with and without osteoporosis [33, 34]. Therefore, the fact that the REMS approach shows excellent values of sensitivity and specificity above 90% by employing a single threshold, which is the same T-score = −2.5 employed by DXA, suggests that the proposed non-ionizing method could be effectively used also in men to classify the patients into diagnostic categories, without requiring confirmatory DXA scans. Analogous results were obtained when sensitivity and specificity were evaluated on two subgroups of subjects: patients aged “under 65 years” and “over/equal to 65 yeas”. In particular for both femur and lumbar spine, the sensitivity and the specificity values showed a marked increase when the 0.3 T-score tolerance was considered for both of the subgroups of subjects due to the high presence of borderline cases.

Furthermore, the high sensibility (equal to 100%) obtained for the femoral site for the subjects aged under 65 years indicates that there are few osteoporotic cases among the considered population and that the REMS technology is able to correctly identify all the osteoporotic men. The diagnostic concordance between DXA and REMS when all the three possible classification categories (osteoporotic, osteopenic, healthy) were considered, resulted to be 82.7% for lumbar spine and 81.8% for femoral neck. Similarly, accepting a 0.3 tolerance on T-score value of borderline cases, the diagnostic concordance reached 92.3% and 95.7% for lumbar spine and femoral neck, respectively. Moreover, one of the parameters that best emphasizes the accuracy of the REMS technology is the Pearson’s correlation coefficient (r), that reached *r* = 0.91 (*p* < 0.0001) for lumbar spine and *r* = 0.90 (*p* < 0.0001) for femoral neck. This finding indicates that the T-score values estimated by REMS are highly correlated with the values of BMD provided by DXA, documenting the existence of a substantial equivalence between the two measurement techniques.

Furthermore, the short-term precision determined at the lumbar spine and femoral neck in the present study with the REMS approach resulted to be better than the corresponding values typically reported in literature for the gold standard. For instance, referring to lumbar spine, in this study the intra-operator RMS-CV (short-term precision) was 0.40% for REMS, whereas it has been reported to be in the range of 1.07–1.34% for DXA [35]. Analogously, for the femoral neck, a typical reported value of intra-operator RMS-CV for DXA is 1.47% [36], whereas REMS resulted in RMS-CV = 0.34%.

Interestingly, the precision accuracy in the male population obtained in this study was within a similar range of previously obtained results in the female population [22], hinting at a high intrinsic accuracy of this ultrasound approach.

However, to the best of our knowledge, there are no studies actually reporting the inter-operator variability of BMD measurements obtained through anteroposterior DXA measurements on male subjects, but only in females where the error account for 1.6% [37].

The great advantage of this technology relies on the fact that it is not affected by age-related degenerative phenomena being able to recognize and automatically exclude artifacts (prostheses, calcifications, osteophytes, metal structures, bone deformities etc.) [13, 38, 39]. These evaluations might have important implications for both the early diagnosis of osteoporosis in young men and in the elderly population who might benefit from the REMS approach.

## Conclusion

Precision, inter-operator repeatability and diagnostic accuracy of REMS investigations were assessed in a male population, in comparison with DXA outcomes, in a multicenter clinical context, taking also into account possible errors in the performed DXA and REMS scans.

Obtained results showed that, when both DXA and REMS investigations were carried out in the strictest compliance with the corresponding guidelines and recommendations, REMS-measured T-score values derived from BMD measurements resulted in good agreement with the corresponding DXA-measured T-score values for each considered anatomical site. This was also coupled with excellent results in terms of short-term precision and inter-operator repeatability.
